# Tumor-Activated TCR**γ** **δ** ^+^ T Cells from Gastric Cancer Patients Induce the Antitumor Immune Response of TCR**α** **β** ^+^ T Cells via Their Antigen-Presenting Cell-Like Effects

**DOI:** 10.1155/2014/593562

**Published:** 2014-03-31

**Authors:** Chaoming Mao, Xiao Mou, Yuepeng Zhou, Guoyue Yuan, Chengcheng Xu, Hongli Liu, Tingting Zheng, Jia Tong, Shengjun Wang, Deyu Chen

**Affiliations:** ^1^Department of Nuclear Medicine, The Affiliated Hospital of Jiangsu University, 438 Jiefang Road, Zhenjiang 212001, China; ^2^Institute of Oncology, The Affiliated Hospital to Jiangsu University, 438 Jiefang Road, Zhenjiang 212001, China; ^3^Department of Immunology and Laboratory Immunology, School of Medical Science and Laboratory Medicine, Jiangsu University, 301 Xuefu Road, Zhenjiang 212013, China

## Abstract

Human **γδ** T cells display the principal characteristics of professional antigen-presenting cells (APCs), in addition to playing a vital role in immunity through cytokine secretion and their cytotoxic activity. However, it is not clear whether **γδ** T cells perform APC-like functions under pathological conditions. In this study, we showed that, in contrast to peripheral-derived **γδ** T cells directly isolated from PBMCs of gastric cancer patients, tumor-activated **γδ** T cells not only killed tumor cells efficiently but also strongly induced primary CD4^+^ and CD8^+^ **αβ** T cells proliferation and differentiation. More importantly, they abrogated the immunosuppression induced by CD4^+^CD25^+^ Treg cells and induced the cytotoxic function of CD8^+^ **αβ** T cells from patients with gastric cancer. In conclusion, tumor-activated **γδ** T cells can induce adaptive immune responses through their APC-like functions, and these cells may be a potentially useful tool in the development of tumor vaccines and immunotherapy.

## 1. Introduction

*γδ* T cells are a distinct subset of CD3^+^ T lymphocytes characterized by the presence of T cell receptors (TCRs), which are encoded by V*γ*- and V*δ*-gene segments [1]. In human peripheral blood, *γδ* T cells typically represent only 3–5% of all T lymphocytes and are V*δ*2^+^ *γδ* T cell subset predominant; however, they are common in the organs and mucosa, and, here, they are V*δ*1^+^ *γδ* T cell subset predominant, acting as the first defense system against the entry of foreign organisms. In contrast to conventional *αβ* T cells, *γδ* T cells express a limited repertoire of TCR V-region genes. Stimulated *γδ* T cells undergo activation, which results in a plethora of poorly defined changes, including proliferation, proinflammatory cytokine, and chemokine secretion, and altered cell surface phenotypes [1]. *γδ* T cells participate in the immune response by direct cytolysis, development of memory phenotypes, and modulation of immune cells, and they have been implicated in autoimmune disorders, immune deficiencies, infections, and tumor diseases.

*γδ* T cells recognize and kill a range of tumor cells with multiple tissue origins [2, 3], and the genetic absence of *γδ* T cells rendered mice significantly more susceptible to tumor growth in vivo [4–6]. The antitumor properties of *γδ* T cells have been exploited as a potential target for tumor immunotherapy [2, 7]. It has been reported that the most common subtype of these cells in human blood is V*γ*9V*δ*2, which recognizes a group of nonpeptide phosphoantigens (PAg)—of which isoprenyl pyrophosphate (IPP) has been well characterized—that are known to be upregulated in infection or cancer [8, 9]. Further, PAgs may be presented to *γδ*-TCRs via a surface molecule (i.e., F1-ATPase) in a manner somewhat analogous to MHC-mediated antigen presentation [10], suggesting that V*γ*9V*δ*2 cells can function as professional antigen-presenting cells (APCs) [11–13]. Human *γδ* T cells exhibit a potent cytotoxicity against various tumor cells as cytotoxic T cells [2, 14–17]. However, the significance of *γδ* T cells expressing the APC-like phenotype and the mechanisms by which they fight tumor cells remains largely unknown. In this study, we showed that *γδ* T cells from patients with gastric cancer could not only serve as targets for *γδ* T-mediated antitumor activity but also display the APC-like phenotype and functions.

## 2. Materials and Methods

### 2.1. Patient Subjects

Human peripheral blood and fresh tumor tissue samples were obtained from gastric cancer patients (16 men and 4 women; age: 47–69 years; median age: 58.1 ± 6.4 years) newly diagnosed on the basis of clinical history, gastroscopic examination, and pathological diagnosis. Healthy controls (8 men and 2 women; age: 39–63 years; median age: 54.4 ± 8.7 years) were also enrolled, based on normal results from laboratory and physical examinations. Ethics approval for this study was granted by the Ethics Committee of the Affiliated Hospital of Jiangsu University, and written informed consent was obtained from all patients enrolled.

### 2.2. Flow Cytometric Assays

Cells (1 × 10^5^) were suspended in PBS containing 2% FBS for 10 min to block nonspecific binding sites and then were incubated at 4°C for 30 min to determine the percentages of subsets of lymphocyte cells with a combination of antibodies as follows: CD3-APC (UCHT1), CD8-PE (B9.11), CD4-FITC (13B8.2), CD80-FITC (MAB104), CD83-PE (HB15a), CD86-PE (HA5.2B7), HLA-DR-PE (IM0464), CD25-PE (B1.49.9), pan *γδ*-PC5 (IMMU510), and CD45-APC (J33); all were purchased from Beckman Coulter. Cell apoptosis was stained with the Apoptosis Detection Kit I (BD Pharmingen) according to the manufacturer's instructions. For indirect staining, cells were washed twice with PBS and then incubated for 20 min at 4°C with PE-conjugated goat anti-mouse IgG. Compensation was set up with single-stained samples; low forward scatter elements (RBC and debris) were excluded from analysis, and 10,000 events were collected and analyzed by FACSAria cytometer (BD Biosciences).

### 2.3. Cell Isolation and Purification

Fresh peripheral blood was collected in sodium-heparin vacutainer tubes. Periphery mononuclear cells (PBMCs) were isolated by Ficoll density gradient (Sigma Aldrich) centrifugation. CD8^+^ T, TCR*γδ* ^+^ T, CD4^+^CD25^−^ T, and CD4^+^CD25^+^ Treg cells were isolated and purified from fresh PBMCs of patients with gastric cancer by magnetic cell separation. In brief, CD8^+^ T (>95% purity) and TCR*γδ* ^+^ T cells (>95% purity, named as peripheral-derived *γδ* T cells) were firstly separated by positive selection using human blood TCR*γδ* ^+^ T and CD8^+^ T Cell Isolation Kits (Miltenyi Biotec) according to the manufacturer's instructions. From the fraction of remaining cells further isolation of CD4^+^CD25^−^ T cells (>90% purity) and CD4^+^CD25^+^ T cells (>95% purity) was accomplished by negative selection and positive selection using a human CD4^+^CD25^+^ Regulatory T Cell Isolation Kit (Miltenyi Biotec).

### 2.4. Generation of Tumor-Activated *γδ* T Cells

Gastric cancer tissues were minced and digested with a triple enzyme mixture comprising collagenase type IV, hyaluronidase, and deoxyribonuclease for 2 h at room temperature. After digestion, the cells were washed twice in RPMI 1640 and then irradiated (30 Gy) and preserved. Peripheral-derived *γδ* T cells (6 × 10^5^ cells/mL) were then cocultured with the irradiated tumor tissue cells (3 : 1 ratio) in RPMI 1640 containing 10% human serum supplemented with l-glutamine, 2-mercaptoethanol, IL-2 (200 U/mL; R&D Systems), and IL-15 (20 ng/mL; R&D Systems) for generation and expansion of tumor-activated *γδ* T cells.

### 2.5. Proliferation Assay of *γδ* T Cells

Irradiated (30 Gy) PBMCs or tumor tissue cells (2 × 10^4^ cells/well) seeded in 96-well plates with 200 *μ*L RPMI 1640 medium containing 10% FBS and IL-2 (100 U/mL) were added to purified autologous *γδ* T cells (6 × 10^4^ cells/well) and incubated at 37°C 5% CO_2_ for 3 days. Cells were pulsed with 1 *μ*Ci/well of [^3^H]TdR and harvested after 12 h. The incorporation of [^3^H]TdR was determined using a liquid scintillation counter (LS6500; Beckman Coulter, Brea, CA, USA).

### 2.6. In Vitro Functional Assay

To determine the functional effect of tumor-activated *γδ* T cells on adaptive immune T cells, an in vitro functional assay was performed as previously described [18]. In brief, autologous CD4^+^CD25^−^ T cells or CD8^+^ T cells (1 × 10^6^ cells/mL) were labeled for 15 min with 4.5 *μ*M carboxyfluorescein succinimidyl ester (CFSE; Sigma Aldrich). Labeled CD4^+^ or CD8^+^ T cells (2 × 10^5^ cells/mL) were cocultured with *γδ* T cells alone or together in the indicated ratios in 24-well plates containing 10% FBS-RPMI 1640 medium at 37°C in 5% CO_2_. To determine the functional effect of the tumor-activated *γδ* T cells on CD4^+^CD25^+^ Treg cells, autologous CD4^+^ T cells (2 × 10^5^ cells/mL) were cocultured with CD4^+^CD25^+^ Treg cells (2 × 10^5^ cells/mL) in the absence or presence of *γδ* T cells, anti-CD3 (OKT3; eBioscience), and anti-CD28 (CD28.2; eBioscience). Proliferation of CD4^+^ or CD8^+^ T cells was determined by fluorescence correlation microscopy (FCM) to assess CFSE dilution on day 3. Transwell experiments were also performed with 24-well plates with a pore size of 0.4 *μ*m (Corning Costar, Cambridge, MA, USA). To determine whether the effect of tumor-activated *γδ* T cells could be blocked by specific antibodies, T cell activity was assessed in the absence or presence of various antibodies such as those against IL-1*β* (AF-201-NA; R&D Systems), IL-6 (AF-206-NA; R&D Systems), IL-12 (MAB1510; R&D Systems), CD80 (MAB140; R&D Systems), CD86 (MAB141; R&D Systems), and IFN-*γ* (AB-285-NA; R&D Systems).

### 2.7. Cytotoxic Assay

The cytotoxic activity of effector T cells was determined by measuring the amount of lactate dehydrogenase (LDH) released from target cells. The commercial LDH Cytotoxicity kit (Beyotime, China) was used according to the manufacturer's instructions. Maximum LDH release of target cells was determined by lysing target cells for 45 min (lysis buffer provided within the assay) and subsequently measuring the LDH from the culture medium. Absorbance values after the colorimetric reaction were measured at 490 nm with a reference wavelength of 655 nm, using a Bio-Rad Model 550 microplate reader (Bio-Rad, Hercules, CA, USA).

### 2.8. Measurement of Cytokines by ELISA

The supernatants from *γδ* T cells culture were collected and stored at −80°C until analysis. Cytokines of IFN-*γ*, IL-1, IL-4, IL-6, IL-10, and IL-12 concentrations were measured using commercial enzyme-linked immunosorbent assay (R&D systems) according to the manufacturer's instructions.

### 2.9. Confocal Microscopy

Tumor cells were labeled with CFSE and incubated with tumor-activated *γδ* T cells stained with mouse antihuman HLA-DR-PE-Cy5 before analysis. Images were captured using confocal microscopy (Leica TCS SP5; Leica Microsystems, Bannockburn, IL, USA) using the LAS AF confocal software. Protein antigen capture between cell-cell interactions was visualized using time-lapse confocal microscopy as described previously [19].

### 2.10. Statistical Analysis

Unless indicated otherwise, data are expressed as mean ± SD. Comparison between two groups was performed by Student's *t*-tests. Data from more than two groups were compared using one-way ANOVA with the Tukey-Kramer multiple comparison test. *P* < 0.05 was considered statistically significant.

## 3. Results

### 3.1. Tumor-Activated *γδ* T Cells Display Characteristics of APCs

**Figure 1:**
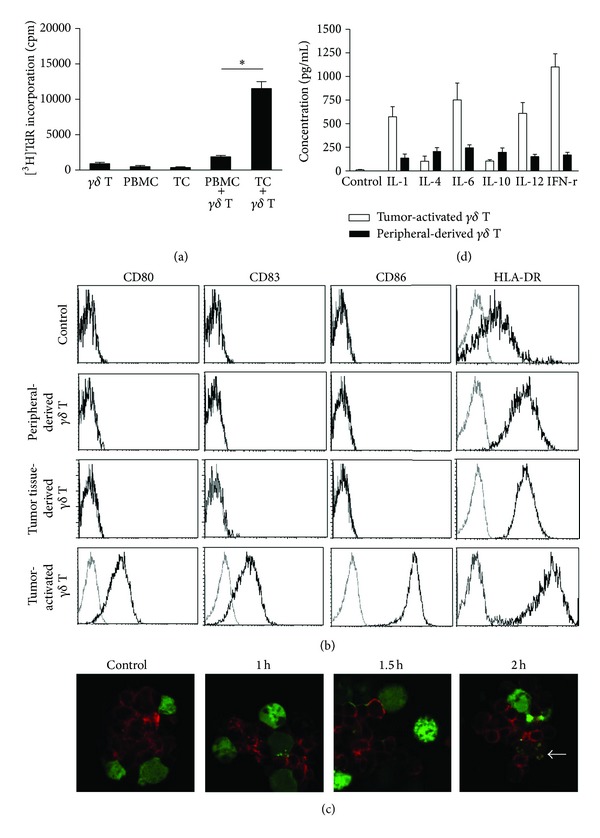
APC-like characteristics of tumor-activated *γδ* T cells. (a) The proliferation of *γδ* T cells was analyzed from [^3^H] thymine deoxyriboside (TdR) incorporation. Irradiated (30 Gy) PBMCs or tumor tissue cells (2 × 10^4^ cells/well) were cocultured with peripheral-derived *γδ* T cells (6 × 10^4^ cells/well) from patients with gastric cancer in 96-well plates for 3 days in the presence of IL-2 (200 U/mL) and IL-15 (20 ng/mL). [^3^H]TdR incorporation was measured during the last 12 h of the incubation. Results are expressed as means ± SD (cpm) of three wells. The data are representative of at least nine independent experiments. (b) FCM was used to analyze gating for the CD3^+^TCR*γδ* ^+^ cell population; phenotypes including CD80, CD83, CD86, and HLA-DR on the *γδ* T cells from PBMCs of healthy donors (control), peripheral-derived, tumor tissue-derived, and tumor-activated *γδ* T cells. Results are representative of nine independent experiments. (c) Uptake and sorting of soluble proteins in tumor-activated *γδ* T cells. *γδ* T cells were cocultured with CFSE-prelabeled tumor cells (green fluorescence) for the indicated time. The cells were then collected, cytocentrifuged, and stained with TCR*γδ*-PECY5 mAb (red fluorescence). The interaction between *γδ* T cells and tumor cells was observed using confocal microscopy. The arrow shows the colocalization of the TCR of *γδ* T cells with some components of tumor cells. Results are representative of three independent experiments. (d) Tumor-activated or peripheral-derived *γδ* T cells (1 × 10^6^ cells/mL) were cultured in RPMI 1640 containing IL-2 (200 U/mL) and IL-15 (20 ng/mL) for 12 h, and the culture supernatants were collected and assayed for IFN-*γ*, IL-1, IL-4, IL-6, IL-10, and IL-12 using ELISA. Data are representative of five independent experiments. *P < 0.01.

*γδ* T cells were directly isolated from the peripheral blood of gastric cancer patients (defined as peripheral-derived *γδ* T cells) and cultured with irradiated autologous PBMCs in the presence of IL-2 and IL-15. The results showed no obvious proliferation of the peripheral-derived *γδ* T cells. In contrast, when the peripheral-derived *γδ* T cells were cultured with irradiated autologous tumor cells in the presence of IL-2 and IL-15, they showed markedly increased proliferation (defined as tumor-activated *γδ* T cells) (Figure 1(a); *n* = 9, *P* < 0.01).

To evaluate the potential functions of tumor-activated *γδ* T cells, we assessed the pattern of surface expression of immune-costimulatory molecules and the profile of cytokines secreted. The results showed no detectable expression of CD80, CD83, or CD86 on the surface of *γδ* T cells from PBMCs of normal donors (control), peripheral-derived *γδ* T cells, and *γδ* T cells directly isolated from tumor tissues (defined as tumor tissue-derived *γδ* T cells). In addition, the antigen-presenting molecule HLA-DR was strongly expressed on *γδ* T cells, and the expression in cells from patients was higher than that in cells from normal donors. Interestingly, we found that the T cell costimulatory molecules CD80 and CD86 and the mature molecule CD83 were substantially upregulated on the tumor-activated *γδ* T cells, along with the obvious increased expression of HLA-DR (Figure 1(b); *n* = 9). To explore the phenomenon that the factors from tumor tissue alone were particular to induce APC-like phenotypes in autogenetic *γδ* T cells, a group by using normal *γδ* T cells activated by tumor tissue was set up. We found that the *γδ* T cells proliferated but lacked changes of APC-like phenotypes (similar to control), suggesting that the *γδ* T cell proliferation was caused by an allogeneic response. Taking together, the factors from tumor tissue alone could contribute to induce APC-like phenotypes in autogenetic *γδ* T cells.

Confocal microscopy results showed that, when *γδ* T cells from patients were cocultured with autologous tumor cells for 2 h, the antigen component from the tumor cells was colocalized on the *γδ* T cells (Figure 1(c); *n* = 3), suggesting that *γδ* T cells could capture some antigens from the tumor cells. Analysis of the profiles of cytokines in the culture supernatants showed that the tumor-activated *γδ* T cells secreted substantially more Th1-prone cytokines such as IFN-*γ*, IL-1, IL-6, and IL-12, but not IL-4 and IL-10, than peripheral-derived *γδ* T cells did (Figure 1(d); *n* = 5).

**Figure 2:**
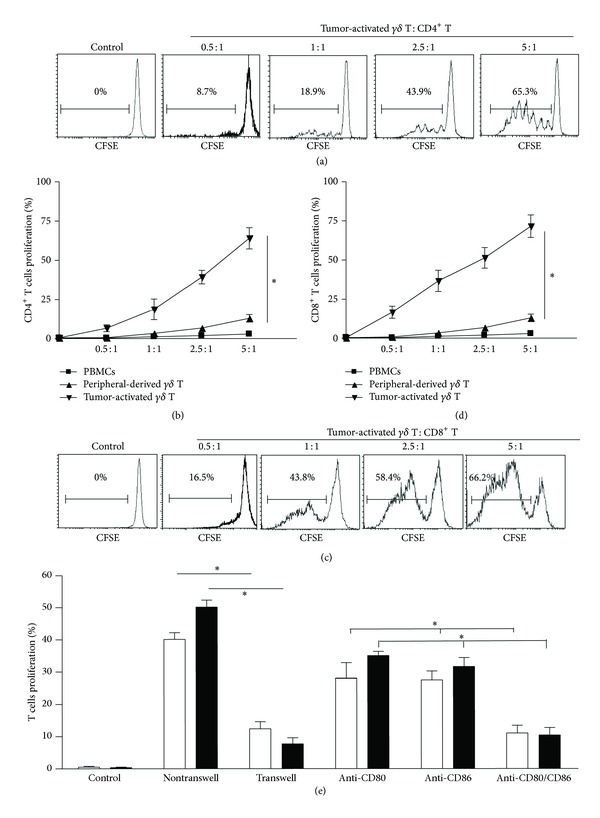
Stimulatory effect of tumor-activated *γδ* T cells on primary CD4^+^ and CD8^+^ T cells. For this test, 2 × 10^5^ cells/mL CFSE-prelabeled primary CD4^+^ T cells ((a) and (b)) or CD8^+^ T cells ((c) and (d)) as responders were incubated with tumor-activated or peripheral-derived *γδ* T cells at the indicated ratios in 24-well plates with or without blocking antibodies (2 *μ*g/mL) and in transwell plates (e). On day 3, the proliferation of CD4^+^ or CD8^+^ T cells was determined by FCM, wherein CFSE dilution was assessed. Results are representative of 3 independent experiments. *P < 0.01.

To investigate the immunostimulatory properties of the tumor-activated *γδ* T cells, the ability to induce expansion of effector CD4^+^ and CD8^+^ *αβ* T cells was examined using in vitro functional assay. The results showed that, in contrast to peripheral-derived *γδ* T cells, tumor-activated *γδ* T cells induced significantly higher proliferation of CD4^+^ T cells (Figures 2(a) and 2(b); *n* = 3, *P* < 0.01) and CD8^+^ T cells (Figures 2(c) and 2(d); *n* = 3, *P* < 0.01) in a number-dependent pattern, as assessed by the reduction in CFSE signals. Further, the proliferation of CD4^+^ or CD8^+^ T cells induced by the tumor-activated *γδ* T cells was blocked in a transwell experiment and anti-CD80/CD86 antibody blocking experiment (Figure 2(e); *n* = 3, all *P* < 0.01), suggesting that the effect of tumor-activated *γδ* T cells on CD4^+^ or CD8^+^ T cells is dependent on cell-cell contact and costimulatory molecule CD80/CD86.

### 3.2. Tumor-Activated *γδ* T Cells Abrogate Immunosuppression Induced by CD4^+^CD25^+^ Treg Cells

**Figure 3:**
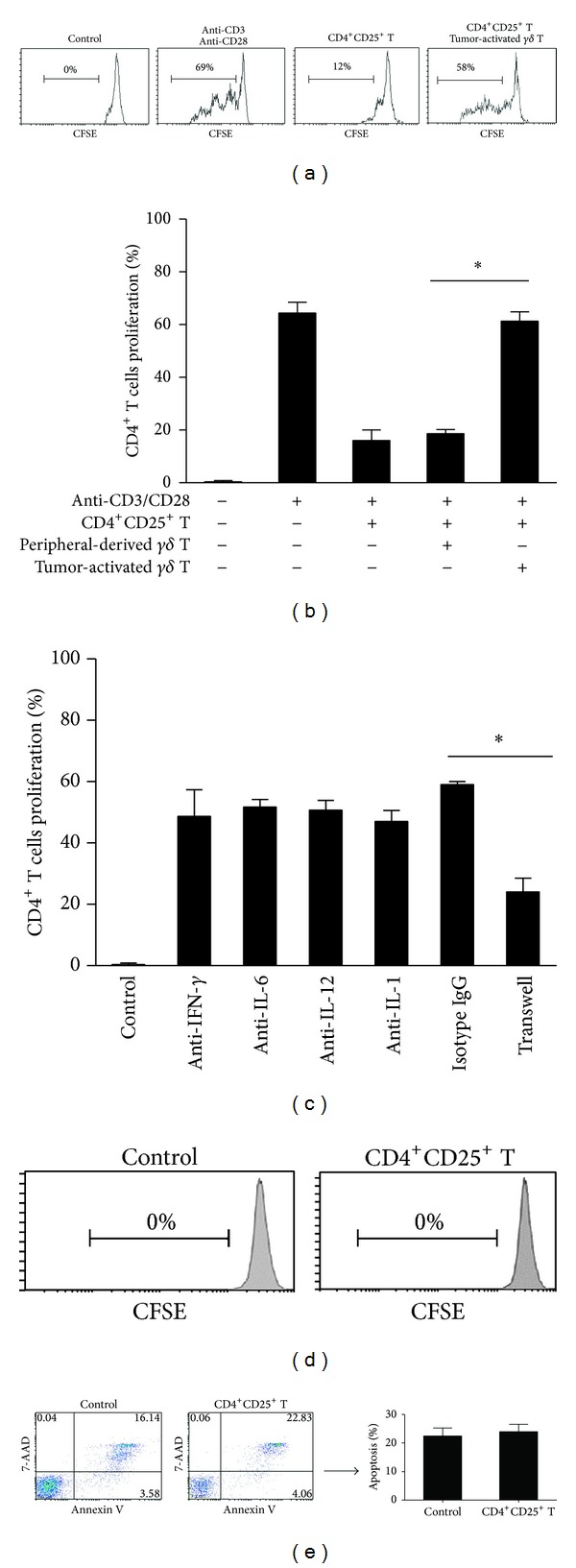
Abrogation of immunosuppressive effect of CD4^+^CD25^+^ Treg cells by tumor-activated *γδ* T cells. ((a) and (b)) Primary CD4^+^ T cells were prelabeled with CFSE as responders, and 2 × 10^5^ cells/mL CFSE-prelabeled primary CD4^+^ T cells were incubated alone or coincubated with autologous CD4^+^CD25^+^ Treg cells at a ratio of 1 : 1 and/or tumor-activated *γδ* T cells at a ratio of 1 : 1 : 3 in the presence of anti-CD3 mAb (1 *μ*g/mL) and anti-CD28 mAb (1 *μ*g/mL) with or without blocking antibodies. The same combinations were also incubated in transwell plates (c) as indicated. The proliferation of CD4^+^ T cells was checked on day 3 by FCM used to assess CFSE dilution. (d) 2 × 10^5^ cells/mL CFSE-prelabeled CD4^+^CD25^+^ Treg cells were incubated alone (control) or coincubated with tumor-activated *γδ* T cells at a ratio of 1 : 1. The proliferation of CD4^+^CD25^+^ Treg cells was checked on day 3 by FCM used to assess CFSE dilution. (e) 2 × 10^5^ cells/mL tumor-activated *γδ* T cells were incubated alone (control) or coincubated with CD4^+^CD25^+^ Treg cells at a ratio of 1 : 1. The apoptosis of CD4^+^CD25^+^ Treg cells was checked on day 2 by FCM. Results are representative of three independent experiments. *P < 0.01.

Our previous study demonstrated that CD4^**+**^CD25^**+**^ Treg cells isolated from tumor patients inhibited the proliferation of autologous effector CD4^**+**^ T cells [20]. To determine the effect of tumor-activated *γδ* T cells on CD4^**+**^CD25^**+**^ Treg cells, the peripheral-derived *γδ* T cells or tumor-activated *γδ* T cells were cocultured with CD4^**+**^CD25^−^ T cells and CD4^**+**^CD25^**+**^ Treg cells, respectively. As shown in Figures 3(a) and 3(b), the proliferation of CD4^**+**^CD25^−^ T cells was inhibited in the presence of CD4^**+**^CD25^**+**^ Treg cells isolated from gastric cancer patients, and it did not change when peripheral-derived *γδ* T cells were added to the culture system. However, when tumor-activated *γδ* T cells were added, the proliferation of CD4^**+**^CD25^−^ T cells was significantly increased (*n* = 3, *P* < 0.01), suggesting that tumor-activated *γδ* T cells, but not peripheral-derived *γδ* T cells, abrogate the immunosuppressive effect induced by CD4^**+**^CD25^**+**^ Treg cells. Further, the above-mentioned effects were blocked in a transwell experiment (Figure 3(c); *n* = 3, *P* < 0.01) but not by specific antibodies against cytokines such as IFN-*γ*, IL-1, IL-6, and IL-12. Further experimental results showed that tumor-activated *γδ* T cells neither induced the proliferation of CD4^**+**^CD25^**+**^ Treg cells (Figure 3(d); *n* = 3) nor enhanced the apoptosis of CD4^**+**^CD25^**+**^ Treg cells (Figure 3(e); *n* = 3), when cocultured with CD4^**+**^CD25^**+**^ Treg cells.

### 3.3. Tumor-Activated *γδ* T Cells Not Only Directly Kill Tumor Cells but Also Activate the Cytotoxic Effects of Autologous CD8^+^ T Cells

**Figure 4:**
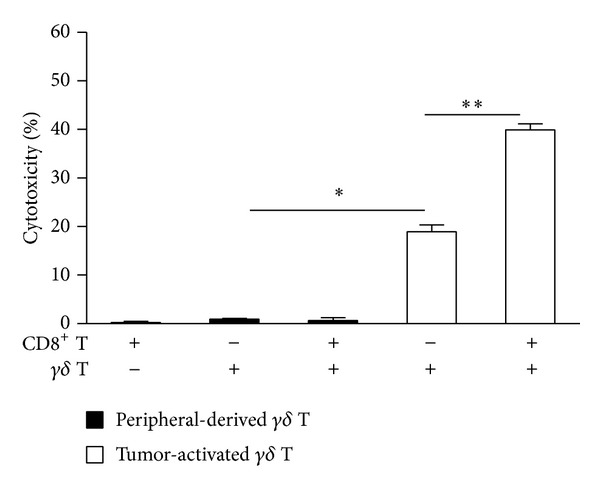
Tumor-activated *γδ* T cells trigger the cytotoxic effect of autologous CD8^+^ T cells. Autologous CD8^+^ T cells (2 × 10^5^ cells/mL) were cocultured with tumor cells in a 24-well plate for 6 h in the absence or presence of peripheral-derived *γδ* T cells or tumor-activated *γδ* T cells at a ratio of 1 : 1 : 1. The culture supernatants were collected and the cytotoxic activity of effector cells was measured using an LDH assay. Results are expressed as means ± SD and are representative of three independent experiments. *P < 0.01, **P < 0.05.

To determine the ability of tumor-activated *γδ* T cells to kill tumor cells and its role in the cytotoxicity of effector CD8^+^ T cells, we examined the cytolysis of tumor-activated *γδ* T cells and primary autologous CD8^+^ T cells incubated with tumor-activated *γδ* T cells. The results showed that tumor cells were not lysed by peripheral-derived *γδ* T cells, CD8^+^ T cells alone, or CD8^+^ T cells cocultured with peripheral-derived *γδ* T cells. Conversely, tumor-activated *γδ* T cells could lyse tumor cells (Figure 4; *n* = 3, *P* < 0.01), and cytotoxicity for the tumor cells was increased in the presence of CD8^+^ T cells (*P* < 0.05), suggesting that tumor-activated *γδ* T cells not only directly kill tumor cells but also activate the cytotoxicity of CD8^+^ T cells.

## 4. Discussion

In general, APCs such as dendritic cells (DCs) sample Ags from target cells or pathogens by phagocytosis and then present or cross-present processed Ags to MHC class II I molecules, to trigger adaptive immune responses by the release of cytokines, expression of costimulatory molecules, and Ag stimulation [12]. On the one hand, *γδ* T cells display characteristics of adaptive immunity, wherein they express a rearranged TCR receptor, generate immunologic memory, and transform into cytotoxic T lymphocytes. On the other, they may also be considered part of the innate immune system, since they respond rapidly to antigenic stimuli, have limited TCR gene usage, and express pattern-recognition receptors [13]. To our knowledge, the present study is the first to identify an APC-like function for *γδ* T cells in tumor patients.

Previous studies demonstrated that human *γδ* T cells from tonsillar tissue have APC functions, efficiently cross-presenting soluble proteins to effector CD8^+^ *αβ* T cells and inducing effector cells differentiation and activation [1, 11, 21]. However, these results were acquired using *γδ* T cells stimulated by the isoprenoid metabolite isopentenyl pyrophosphate (IPP) in vitro, and whether *γδ* T cells function like APCs under different disease circumstances, particularly tumors remained largely unknown. In the present study, peripheral-derived *γδ* T cells from gastric cancer patients were used. Our findings showed that these peripheral-derived *γδ* T cells were activated and proliferated when stimulated by autologous tumor cells in vitro, suggesting that tumor cells were equipped with the signals to activate peripheral-derived *γδ* T cells. The underlying mechanism is not clear at present. We previously found that that autologous tumor cells selectively expanded *γδ* T cells among CD4^−^CD8^−^ PBMCs from cancer patients and this phenomenon was related to TCR and NKG2D signals.

Accumulating experimental and clinical data indicate that *γδ* T cells can recognize aminobisphosphonates and phosphorylated intermediates of the bacterial nonmevalonate isoprenoid pathway, known as phosphoantigens. In addition, high concentrations of IPP, possibly generated because of a dysregulated mevalonate pathway as well as ectopically expressed mitochondrial F1-ATPase/apolipoprotein I complex on malignant cells, can selectively induce *γδ* T cell expansion through the TCR pathway [3, 8, 22]. NKG2D recognizes the stress-inducible MHC class I-related chains A and B (MIC A/B) and glycophosphatidylinositol-linked proteins UL16-binding proteins (ULBPs), which are expressed by many tumor cells [23–26]. The engagement of NKG2D provides a costimulatory signal for *γδ* T cell activation, allowing for the amplification of TCR-mediated priming upon recognition of ligand(s) on tumor cells.

The tumor-activated *γδ* T cells showed APC-like characteristics in terms of phenotype, cytokine profile, and functions. (i) They strongly expressed HLA-DR and the costimulator molecular CD80/CD86 and secreted the proinflammatory cytokines IL-1, IL-6, IL-12, and IFN-*γ*. (ii) They triggered the proliferation and differentiation of primary CD4^+^ or CD8^+^ *αβ* T cells and inhibited the function of CD4^+^CD25^+^ Treg cells. (iii) Most importantly, in addition to their direct cytotoxicity, they activated CD8^+^ *αβ* T-mediated cytotoxicity to tumor cells. These phenotypic and functional features are consistent with those found previously [11, 19, 27]. However, our understanding of the APC effects of *γδ* T cells on *αβ* T cell differentiation is currently rudimentary. Our results showed that peripheral-derived *γδ* T cells have no APC-like functions, which exclude their involvement in the control of *αβ* T cell responses in patients with gastric cancer. In contrast, tumor-activated *γδ* T cells behave like APCs by the rapid acquisition of APC characteristics in the activation of peripheral-derived *γδ* T cells, in a manner reminiscent of mature dendritic cells (DCs). The responses induced by the *γδ* T cells were potent, and they induced robust proliferation responses among primary autologous CD4^+^ and CD8^+^ *αβ* T cells, possibly through an unknown antigen-presenting pathway, proinflammatory cytokines, and costimulator molecules. These functions are highly beneficial, and *γδ* T cells are a unique and conserved population of lymphocytes that have been the subject of a recent explosion of interest owing to their essential contributions to many types of immune responses and immunopathology [28]. The functional difference between peripheral-derived and tumor-activated *γδ* T cells is ascribed to the suppressive state of the immune system in cancer patients.

Considerable attention in immunotherapy research is currently focused on human *γδ* T cells because of their functional uniformity [29]. The role of *γδ* T cells in oncology is concentrated around their potential applications in cancer treatment, for direct cytolysis. In terms of cellular immunotherapy, it may be important to emphasize that *αβ* T cell differentiation induced by *γδ* T cells leads to CD4^+^ T helper cell and effector CD8^+^ T cell responses. Further, experimental and clinical data indicate that *γδ* T cells exhibit a potent HLA-unrestricted lytic activity against various tumor cell lines and display antitumor effects [17, 30, 31]. In addition to TCR-dependent recognition, activation of the killer receptor NKG2D is involved in the cytotoxic activity of *γδ* T cells. In general, the cytotoxicity of *γδ* T cells to tumor cells involves the TCR and NKG2D receptors and depends on the perforin/granzyme pathway.

Our results also suggested that tumor-activated *γδ* T cells directly lyse the target tumor cells and trigger the activation and functioning of CD4^+^ and CD8^+^ T cells. Of note, the molecular mechanisms underlying *γδ* T cell-mediated activation of conventional CD4^+^ or CD8^+^ *αβ* T cells are similar to those employed by professional APCs, which involve TCR signals and the costimulatory molecules CD80/CD86. Therefore, a possible mechanism for the APC-like behavior of tumor-activated *γδ* T cells is that these cells are loaded with tumor components for a brief period, during which they process and express costimulatory molecules CD80/CD86. The recognition of tumor cells by *γδ* T cells does not depend on MHC-mediated Ag presentation, which may represent a key advantage in immunotherapy during advanced stages of cancer [32].

It is well established that the aggregation of CD4^+^CD25^+^ Treg cells in the tumor milieu is involved in the immune escape of tumors and is detrimental for immunotherapy among tumor patients [20, 33, 34]. Further, an interaction between CD4^+^CD25^+^ Treg cells and *γδ* T cells has been reported [35]. Recent data have further shown that *γδ* T cells from tumor tissues are positively correlated with Foxp3^+^ suppressive T cells in advanced breast tumors and inversely correlated with relapse-free and overall survival of breast cancer patients [36]. These findings raise interesting questions on whether tumor-activated *γδ* T cells are capable of weakening CD4^+^CD25^+^ Treg cell-mediated immunosuppression. Our results demonstrated an additional value to the proposed immunotherapy with *γδ* T cells, wherein the immune suppression by CD4^+^CD25^+^ Treg cells can be overcome since it requires cell-cell contact. The underlying mechanism could be that tumor-activated *γδ* T cells show enhanced stimulation of CD4^+^ T cell proliferation, rather than direct suppression by CD4^+^CD25^+^ Treg cells, since our results indicated that tumor-activated *γδ* T cells failed to induce proliferation or apoptosis of CD4^+^CD25^+^ Treg cells.

From the results, we speculated that tumor-activated *γδ* T cells kill tumor cells and simultaneously present certain Ag(s) from tumor cells. Tumor-antigen signals, together with costimulatory molecules and cytokines, could effectively trigger the activation and functioning of CD4^+^ and CD8^+^ T cells. Further investigation of this hypothesis may show that *γδ* T cells were compared favorably with professional APCs such as DCs with respect to their advantage of expansion in vitro and direct activation by signals preferentially expressed on tumor cells, such as NKG2D ligands MIC A/B and ULBPs [22, 37, 38], and, thus, these cells may become a continual and renewable source of functional APCs. They may be used to produce tumor vaccines to induce an adaptive immune response against tumors. We believe that *γδ* T cells will play a key role in developing cancer immunotherapy strategies.
